# Temporal and spatial distribution of sporotrichosis in the Rio de Janeiro Metropolitan Area, Brazil: a comparison between human and animal cases (2013-2020)

**DOI:** 10.1590/0102-311XEN133024

**Published:** 2025-02-24

**Authors:** Victória Catharina Dedavid Ferreira, Anna Barreto Fernandes Figueiredo, Mônica de Avelar Figueiredo Mafra Magalhães, Sandro Antonio Pereira, Wagner Tassinari

**Affiliations:** 1 Instituto de Saúde Coletiva, Universidade Federal da Bahia, Salvador, Brasil.; 2 Escola Nacional de Saúde Pública Sergio Arouca, Fundação Oswaldo Cruz, Rio de Janeiro, Brasil.; 3 Instituto Nacional de Infectologia Evandro Chagas, Fundação Oswaldo Cruz, Rio de Janeiro, Brasil.; 4 Instituto de Comunicação e Informação Científica e Tecnológica em Saúde, Fundação Oswaldo Cruz, Rio de Janeiro, Brasil.; 5 Instituto de Ciências Exatas, Universidade Federal Rural do Rio de Janeiro, Seropédica, Brasil.

**Keywords:** Sporotrichosis, Zoonosis Surveillance, One Health, Spatial Analysis, Time Factors, Esporotricose, Vigilância de Zoonoses, Saúde Única, Análise Espacial, Fatores de Tempo, Esporotricosis, Vigilancia de Zoonosis, Salud Única, Análisis Espacial, Factores de Tiempo

## Abstract

Cat-transmitted sporotrichosis is currently hyperendemic in the Rio de Janeiro Metropolitan Area, Brazil. Despite the zoonotic context, surveillance is fragmented, with human and animal contagion being assessed separately. This study aimed to describe and compare spatial and temporal patterns of sporotrichosis cases in humans and animals (cats and dogs) reported to the Brazilian Information System for Notificable Diseases in the Rio de Janeiro metropolitan area, from 2013 to 2020. We conducted an ecological study based on the spatial and temporal evolution of sporotrichosis in the area. We compared the time series of human and animal cases per month. We also compared the cumulative human incidences and the ratio of animal cases per inhabitant by neighborhood or subdistrict and explored spatial correlation with global and local Moran’s I. During the period, 9,552 human and 12,532 animal sporotrichosis suspected cases were reported. Via spatial and temporal exploratory analyses, we verified actions that favored notification during this period, such as establishing mandatory notification and campaigns related to public veterinary care. We also verified the existence of clusters in the west zone of the capital and border cities, and the expansion of sporotrichosis to other disadvantaged areas in the capital and the outskirts of the metropolitan area. Moreover, we observed divergent patterns between human and animal sporotrichosis distribution in time and space. Our findings show a spatial expansion of sporotrichosis in humans and animals; however, they also highlight the limitations of ongoing surveillance, indicating we probably are underestimating magnitude of the problem.

## Abstract (pt)

A esporotricose transmitida por gatos é atualmente hiperendêmica na Região Metropolitana do Rio de Janeiro, Brasil. Apesar do contexto zoonótico, a vigilância é fragmentada, sendo as dimensões humana e animal avaliadas separadamente. Este estudo descreve e compara os padrões espaciais e temporais dos casos de esporotricose em humanos e animais (cães e gatos) notificados ao Sistema de Informação de Agravos de Notificação na Região Metropolitana do Rio de Janeiro, no período de 2013 a 2020. Foi realizado um estudo ecológico, baseado na evolução espacial e temporal da esporotricose na área, comparando-se as séries temporais para casos humanos e animais por mês. Também comparamos as incidências humanas cumulativas e a proporção de casos de animais por habitante por bairros ou subdistritos e exploramos a correlação espacial com Moran global e local. Durante o período, foram notificados 9.552 casos suspeitos de esporotricose humana e 12.532 de animais. Análises exploratórias espaciais e temporais apontaram ações que favoreceram a notificação nesse período, como o estabelecimento da notificação obrigatória e campanhas relacionadas à assistência veterinária pública. Verificou-se também a existência de aglomerados na zona oeste da capital e cidades fronteiriças e a expansão da esporotricose para outras áreas desfavorecidas da capital e da periferia da região metropolitana. Ademais, observamos padrões divergentes entre a distribuição da esporotricose humana e animal no tempo e no espaço. Nossos achados mostram uma expansão espacial da esporotricose para humanos e animais; no entanto, eles também destacam as limitações da vigilância atual, indicando subestimação da magnitude do problema.

## Abstract (es)

La esporotricosis transmitida por gatos es actualmente hiperendémica en la Región Metropolitana de Río de Janeiro, Brasil. A pesar del contexto zoonótico, la vigilancia está fragmentada, y las dimensiones humana y animal se evalúan por separado. Este estudio describe y compara los patrones espaciales y temporales de los casos de esporotricosis en humanos y animales (perros y gatos) notificados al Sistema de Información de Enfermedades de Notificación Obligatoria en la Región Metropolitana de Río de Janeiro, en el período de 2013 a 2020. Se realizó un estudio ecológico, basado en la evolución espacial y temporal de la esporotricosis en la región, comparando las series temporales de casos humanos y animales por mes. También se compararon las incidencias humanas acumuladas y la proporción de casos de animales por habitante por barrios o subdistritos, y se exploró la correlación espacial con Moran global y local. Durante el período, se reportaron 9.552 casos sospechosos de esporotricosis humana y 12.532 casos en animales. Los análisis exploratorios espaciales y temporales indicaron acciones que favorecieron la notificación en este período, como la notificación obligatoria y las campañas relacionadas con la atención veterinaria pública. También hubo agrupaciones en la zona oeste de la capital y las ciudades fronterizas, y la expansión de la esporotricosis hacia otras zonas desfavorecidas de la capital y la periferia de la región metropolitana. Además, se observaron patrones divergentes entre la distribución de la esporotricosis humana y animal en el tiempo y el espacio. Los hallazgos muestran una expansión espacial de la esporotricosis a humanos y animales; sin embargo, también resaltan las limitaciones de la vigilancia continua, lo que indica una subestimación de la magnitud del problema.

## Introduction

Sporotrichosis is a subcutaneous mycosis caused by a pathogenic fungus species of the *Sporothrix* genus, which affects humans and other mammals ^1^ ^,^ ^2^. Generally, infection occurs via traumatic inoculation of *Sporothrix* sp., found in soil, plants, and decaying organic matter ^3^. However, cat-transmitted sporotrichosis has become the main concern for disease dissemination in Brazil and Latin America, especially because of the emergence of the highly virulent species *Sporothrix brasiliensis* ^3^ ^,^ ^4^. Cats are the most susceptible species to *Sporothrix* sp. and are the main source of this etiological agent for humans and other animals in Brazil ^4^ ^,^ ^5^.

Currently, cat-transmitted sporotrichosis is considered hyperendemic in Rio de Janeiro, Brazil ^6^. The number of cases of the disease started increasing in the state around the late 1990s ^7^ ^,^ ^8^, and has been expanding geographically since then ^6^. Although Rio de Janeiro continues to be the epicenter of the disease, it has already become a national concern described nationwide ^6^ ^,^ ^9^ ^,^ ^10^ ^,^ ^11^ ^,^ ^12^ ^,^ ^13^ ^,^ ^14^ ^,^ ^15^ ^,^ ^16^ ^,^ ^17^, which is expanding to other countries ^3^ ^,^ ^18^ ^,^ ^19^.

In 2013, mandatory notification for human sporotrichosis cases was established in the state of Rio de Janeiro, followed by the recommendation for notification of suspected animal cases in 2014. However, few studies have explored the official surveillance system database ^20^ ^,^ ^21^ ^,^ ^22^. Moreover, studies describing animal cases in Rio de Janeiro are mainly based on data from a public research institution that provides veterinary care ^23^ ^,^ ^24^, but they are probably underrepresenting the current disease situation in the capital and other cities of the metropolitan area ^4^.

Cat-transmitted sporotrichosis control is a challenge due to difficulties in preventing cat-to-cat transmission ^25^. Culturally, cats are often granted outdoor access, facilitating contact with other infected animals and consequently, transmission ^26^. Furthermore, in Brazil access to public or low-cost veterinary care is limited, and sporotrichosis treatment is long and expensive, with frequent disease recurrence ^27^. Therefore, surveillance is key for controlling this disease ^28^ ^,^ ^29^.

Sporotrichosis national expansion is often related to the lack of attention given to the cat population, highlighting the importance of the One Health approach for combating the problem ^3^ ^,^ ^6^. Due to zoonotic transmission, feline, human and canine cases should be spatially and temporally correlated ^6^ ^,^ ^23^ ^,^ ^30^. Hence, analyzing case notifications for humans and animals simultaneously can elucidate patterns that perhaps could not be observed separately.

Thus, this study aimed to analyze and compare spatial and temporal patterns of sporotrichosis cases in humans and animals (cats and dogs) reported to Brazilian Information System for Notificable Diseases (SINAN, acronym in Portuguese), in Rio de Janeiro Metropolitan Area from 2013 to 2020.

## Methods

### Study design

This is an ecological study based on the spatial and temporal evolution of sporotrichosis in humans and domestic animals (cats and dogs), using data from SINAN connected to the Rio de Janeiro State Health Department (SES/RJ, acronym in Portuguese), from 2013 to 2020.

### Data

This study included the record of notifications comprising human sporotrichosis (codes B42 and subcategories from the 10th version of the International Classification of Diseases - ICD-10) and the record of notification comprising animal sporotrichosis in cats and dogs reported via SINAN. Notably, the SINAN form used for animal diseases was initially designed for reporting epizootics, so the terminology is mostly related to outbreaks and not individuals. Cases were selected from notifications reported until September 2021, by date of first symptoms for humans and the “onset date of the outbreak” for animals, from January 2013 to December 2020. Cases were also selected by address for humans and occurrence for animals, including those within the Rio de Janeiro Metropolitan Area.

### Study area

Rio de Janeiro Metropolitan Area is located in southeastern Brazil and is composed of 21 municipalities with a population of 12,688,743 inhabitants ^31^ ^,^ ^32^. The area, which is located between latitude 22º47’31.5” South and longitude 43º09’00.6” West, is the second biggest metropolitan area in Brazil and the third in Latin America ^33^. The capital of the state (the city of Rio de Janeiro) is divided in 160 neighborhoods, while the other municipalities are subdivided into 70 subdistricts in total.

### Ethics statement

This study was approved by the research ethics committees of the Sergio Arouca National School of Public Health/Oswaldo Cruz Foundation (ENSP/FIOCRUZ, acronym in Portuguese) (CAAE 50773321.2.0000.5240) and the Rio de Janeiro’s Research Coordination of the Superintendence of Health Education (COOPES/SUPES, acronym in Portuguese). Necessary measures were taken to preserve data confidentiality and anonymity during processing and analyses.

### Statistical analyses

Cases were organized into monthly time series, following the date of first symptoms (humans) or the onset date of the outbreak (animals), considering if they were confirmed or not. The percentage of confirmed cases by month and species (between humans and animals) were compared. Temporal independence was checked using the Ljung-Box test ^34^. Time series were smoothed using locally weighted regression scatterplot smoothing (LOWESS) ^35^.

Cases were georeferenced by address, using an algorithm developed by the Institute of Scientific and Technological Communication and Information in Health (ICICT/FIOCRUZ - LabGeo), which uses Google Maps for finding coordinates ^36^. Coordinates categorized as “geometric center” and “approximate” (indicating lower accuracy ^37^) were manually reviewed. Points representing each sporotrichosis case were aggregated and counted by neighborhood or subdistrict.

For each spatial unit, the cumulative incidence of human sporotrichosis cases per 10,000 inhabitants and the ratio of animal sporotrichosis cases per 10,000 human inhabitants were calculated. It was chosen to calculate the ratio of animal cases in reference to human inhabitants because there is no available trustworthy estimate of animal population per neighborhood or subdistrict. This strategy has been used before for canine leishmaniasis cases ^38^.

Global Moran’s indexes were estimated to investigate spatial independence, and local Moran’s to identify spatial clusters ^39^. Both were calculated based on a contiguity matrix ^40^ defined by land borders, and islands were attached manually to the closest neighborhoods considering transport options (by road or boat). Local clusters were classified as non-significant or significant. When significant, they were further divided into four categories: high-high (high-value clusters), low-low (low-value clusters), low-high (low-values surrounded by high-values), and high-low (high-values surrounded by low-values).

All statistical analyses were conducted in the R programming language (http://www.r-project.org), using core packages like *stats* and *forecast* ^40^ for time series analyses, and *geobr* ^41^, *sf* ^42^, and *spdep* ^43^ for spatial analyses. A 5% alpha significance level was considered for all statistical inference analyses.

## Results

**Figure 1:**
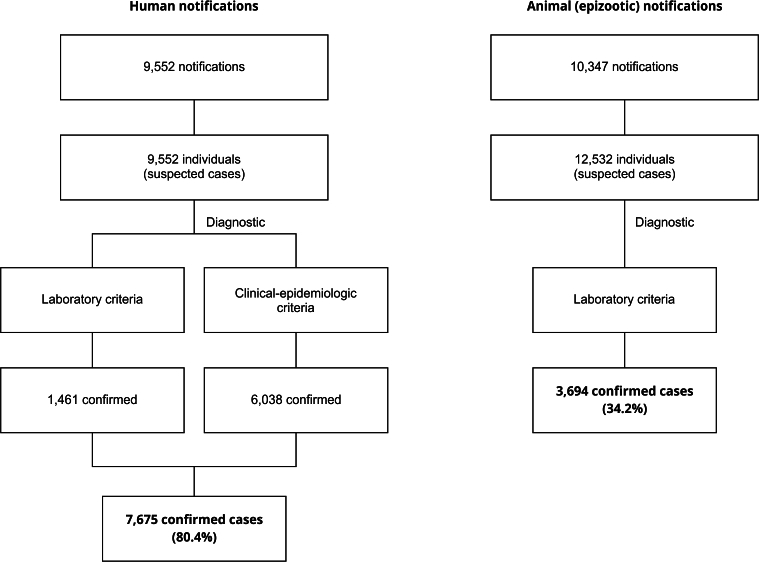
Case definition flowchart for human and animal sporotrichosis notifications and confirmed cases (2013-2020) on the Brazilian Information System for Notificable Diseases (SINAN, acronym in Portuguese).

From 2013 to 2020, 9,552 suspected cases of human sporotrichosis and 12,532 suspected cases of canine and feline sporotrichosis (compiled into 10,347 epizootic notifications) were notified to the state system. Of these, 7,675 human cases (80.4%) were confirmed, either by laboratory or clinical-epidemiological criteria, and 3,795 animal cases (30.3%) were confirmed by laboratory criteria (this is the only option available when reporting animal diseases on the system) (Figure 1). Animal cases included cats (10,791 reported and 3,694 [34.2%] confirmed) and dogs (1,741 reported and 101 [5.8%] confirmed).

### Results for time series

**Figure 2:**
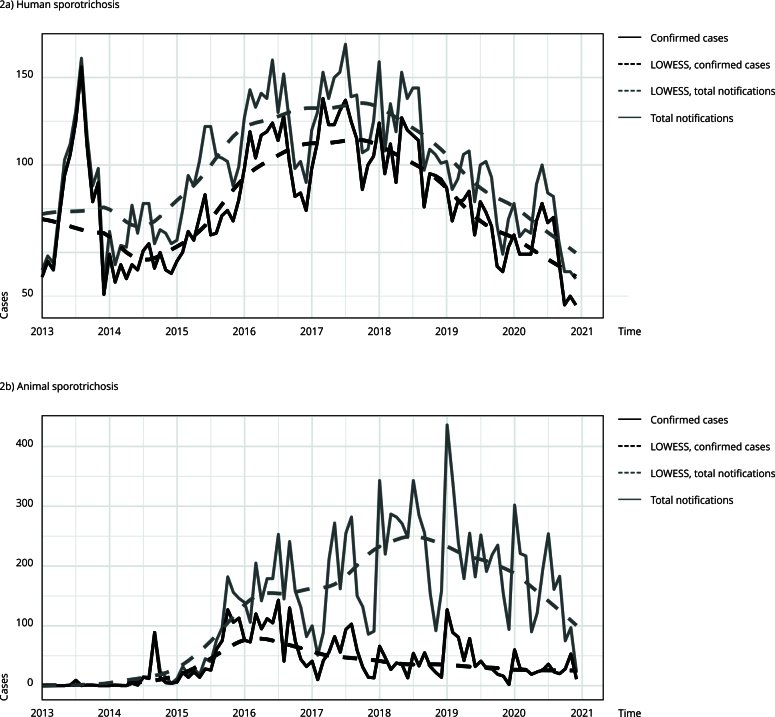
Monthly human and animal sporotrichosis cases, comprising total notifications and confirmed cases, in Rio de Janeiro Metropolitan Area, Brazil (2013-2020). Smoothed LOWESS is shown for total notifications of confirmed cases.

The four time series (human and animal total notifications and confirmed cases) seemed to be autocorrelated in time since all of them presented a significant p-value for the Ljung-Box test. We did not observe any seasonality patterns. Figure 2 shows the plotted raw and smoothed time series of human and animal cases.

In 2013, a brief wave of human cases occurred, coinciding with the start of mandatory notifications for humans. August 2013 was the month with the highest number of confirmed cases (156 of 161 notifications). The number of cases dropped in 2014, then gradually rose to a peak in 2017, with the highest number of notified cases (169). From 2017 to 2020, cases decreased, reaching the lowest number of confirmed cases in October 2020 (20). Overall, the average number of human confirmed cases was 79.2 with a 79.1% average confirmation rate per month (Figure 2a).

Animal sporotrichosis cases started rising in 2014, peaking in the second semester before decreasing. In 2015, cases rose again to nearly 100 per month until 2016 for total notifications and confirmed cases. Until the end of 2016, the difference between total notifications and confirmations was proportional (similar to the time series for human). However, from 2017 to 2020, this difference increased drastically. January 2019 had the highest notifications (436), but only 29.1% of those were confirmed (127). July 2016 had the highest confirmed cases (143 of 253 notifications [56.5%]). Overall, the average of animal confirmed cases was 39.5 with 39.1% average confirmation rate per month (Figure 2b).

By comparing both time series for human and animal confirmed cases, we can observe brief moments of correlation, especially in 2016 and 2017. Higher differences occurred in 2013 and 2014, when fast waves are observed in both series but in different times. Both LOWESS waves seem to increase in 2014 to 2016 for confirmed and notified cases, but for animal cases they decrease soon after their peak. For human cases, the smoothed LOWESS wave starts to decrease only in 2018.

### Results for spatial distribution

**Figure 3:**
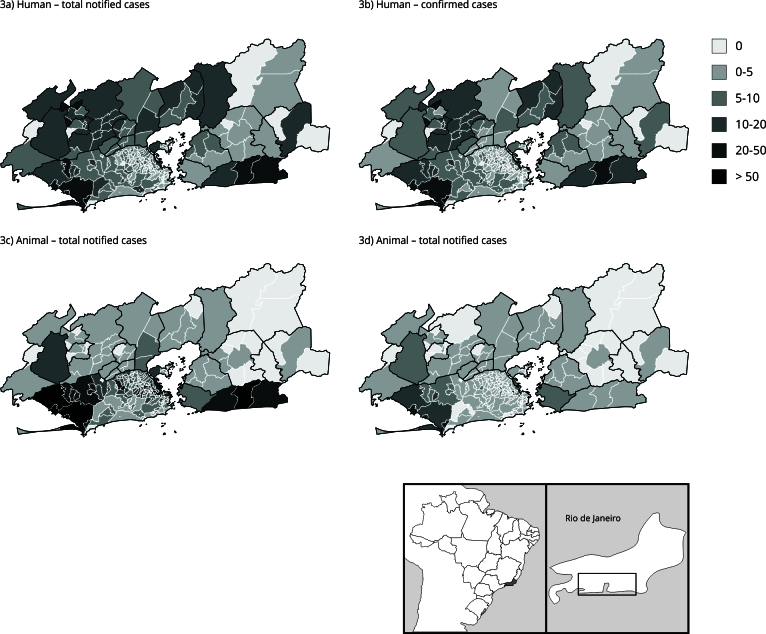
Spatial distribution of human notification cumulative incidence, confirmed human cases cumulative incidence, animal notifications ratio, and confirmed animal cases ratio, all per 10,000 inhabitants, in Rio de Janeiro Metropolitan Area, Brazil (2013-2020).

Figure 3 presents maps showing human sporotrichosis cumulative incidences and animal sporotrichosis ratios per inhabitant, both for notified and confirmed cases, calculated for each spatial unit. Table 1 shows the total number of notifications and confirmed cases for humans and animals per municipality.

**Table 1:** Frequency of total notifications and confirmed cases for sporotrichosis in humans and animals (cats and dogs) per municipality, reported in Rio de Janeiro Metropolitan Area, Brazil (2013-2020).

Municipality	Human sporotrichosis			Animal sporotrichosis		
	Total suspected cases	Confirmed cases		Total suspected cases	Confirmed cases	
	n	n	%	n	n	%
Rio de Janeiro	4,707	4,110	87.3	10,469	2,803	26.8
Nova Iguaçu	1,094	1,041	95.2	94	40	42.6
São Gonçalo	538	482	89.6	281	255	90.7
Duque de Caxias	753	358	47.5	486	226	46.5
Magé	320	287	89.7	42	25	59.5
Belford Roxo	435	264	60.7	48	10	20.8
Maricá	300	249	83.0	523	11	2.1
Queimados	162	151	93.2	10	7	70.0
Mesquita	151	137	90.7	16	7	43.8
São João de Meriti	308	124	40.3	90	28	31.1
Japeri	172	99	57.6	11	7	63.6
Seropédica	93	75	80.6	79	58	73.4
Nilópolis	92	70	76.1	25	10	40.0
Niterói	176	65	36.9	338	296	87.6
Itaguaí	59	52	88.1	39	10	25.6
Itaboraí	62	50	80.6	12	9	75.0
Guapimirim	53	37	69.8	2	1	50.0
Paracambi	52	24	46.2	4	1	25.0
Rio Bonito	44	24	54.5	4	4	100.0
Cachoeiras de Macacu	10	3	30.0	0	0	0.0
Tanguá	5	2	40.0	0	0	0.0

Regarding human sporotrichosis, Barra de Guaratiba had the highest incidence for both notified (61.5 cases per 10,000 inhabitants) and confirmed (55.9) cases, followed by Pedra de Guaratiba (48.5 and 45.3, respectively), both in western Rio de Janeiro city. High incidences were also noted in Sepetiba (30.4 suspected and 28.9 confirmed), Guaratiba (21.5 and 20.8), Paciência (20.6 and 19.0) and Santa Cruz (19.0 and 17.5), all located west of the capital. Vidigal, a *favela* located in the south of Rio de Janeiro city (the wealthiest region), had the fifth highest incidence of confirmed cases (25.8) and the sixth for suspected cases (25.8). Other municipalities with high incidences include Maricá (southeast), especially its central subdistrict (30.7 and 27.3); Japeri (northwest), especially the southern subdistrict (38.0 and 23.5). The lowest incidences occurred mostly in neighborhoods on the north, south, and southwest of Rio de Janeiro city, and in some municipalities located east of Rio de Janeiro Metropolitan Area: Niterói, south of São Gonçalo, Itaboraí, Tanguá, Cachoeiras de Macacu, west and east of Rio Bonito. In the center-north, Duque de Caxias and Nilópolis had lower incidences of confirmed cases.

We observe drastic differences when comparing the maps for ratio of animal cases and incidence of human cases. The two animal ratio maps (confirmed and total cases) also show clear differences between them. Especially regarding animal ratios, there are areas with high total notifications but low confirmed cases. The highest ratios of confirmed and total animal cases were in Praça da Bandeira, northeast of the capital, with much higher values than other locations (202.1 confirmed and 1,345.9 notified animal cases per 10,000 inhabitants). In Maricá, although many cases were notified, few were confirmed. A similar pattern occurred in northeastern Rio de Janeiro and the bordering subdistricts south of Duque de Caxias.

Higher ratios of confirmed animal cases occurred west of the capital, especially in Pedra de Guaratiba (31.6), Sepetiba (28.3), Barra de Guaratiba (22.4), Guaratiba (18.4), Santa Cruz (16.2) and Inhoaíba (11.5). High values were observed in northern neighborhoods, like Benfica (15.2), Mangueira (14.6), and Santo Cristo (12.7). In the metropolitan area, Seropédica and Niterói had notable ratios (7.4 and 6.1 confirmed animal cases per 10,000 inhabitants, respectively). Some subdistricts located northeast and northwest of Rio de Janeiro Metropolitan Area have not notified a single suspected case of animal sporotrichosis during this period. Two subdistricts in Japeri have had high incidences of human cases but did not report any cases of animal sporotrichosis. Vidigal presented one of the highest incidences of human cases, but only one reported animal case that was not confirmed.

The calculated values of Moran’s I global value suggest the existence of spatial dependency for both outcomes: incidence of confirmed human sporotrichosis per 10,000 inhabitants (Moran’s I = 0.29, p-value < 0.01) and ratio of confirmed animal sporotrichosis cases per 10,000 inhabitants (Moran’s I = 0.06, p-value < 0.01).

**Figure 4:**
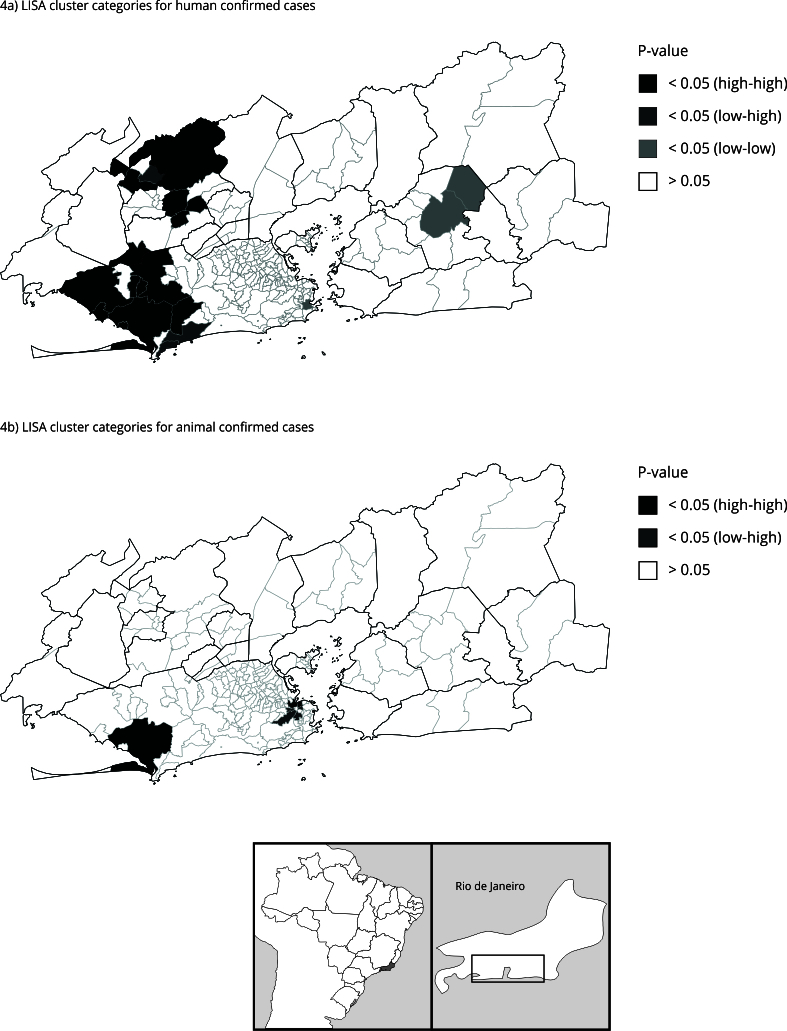
Local Moran classification and p-value for human cumulative incidence and local Moran classification and p-value for animal ratio, all per 10,000 human inhabitants, shown by neighborhood and subdistrict in Rio de Janeiro Metropolitan Area, Brazil (2013-2020).

Figure 4a shows clusters of statistical significance for human incidences. Higher local Moran values are mainly located in west of the capital, forming a high-high significant cluster represented by: Pedra de Guaratiba (12.9), Sepetiba (6.0), Guaratiba (5.2), Santa Cruz (2.5), Cosmos (1.4), Campo Grande (0.32), Inhoaíba (1.2), Vargem Grande (0.77), and the south subdistrict of Nova Iguaçu (0.35). Another significant high-high cluster is located northwest of Rio de Janeiro Metropolitan Area, including the center and north of Nova Iguaçu and west of Japeri. There are two significant low-high clusters, located east of Japeri and southwest of the capital - in neighborhoods with less human density: Recreio dos Bandeirantes (-0.05) and Grumari (-3.6). There are two clusters of low-low incidence: in Botafogo (southeast of the capital, wealthiest area) and Itaboraí (east Rio de Janeiro Metropolitan Area), but with lower Moran values (ranging 0-1.5).

Local Moran indexes for the ratio of animal cases show two significant clusters (Figure 4b). In the same way as we observed for human incidence, there is a high-high cluster west of the capital, however, this one includes only Guaratiba (0.8). Although not significant, other neighborhoods in this area have high local Moran values: Pedra de Guaratiba (2.2), Sepetiba (1.8), and Santa Cruz (0.5). Another high-high cluster is observed east of the capital, including Santo Cristo (1.6) and São Cristóvão (1.0), neighborhoods near the outlier Praça da Bandeira (1.4). There is a significant low-high cluster englobing Rio Comprido (-0.4), Tijuca (-0.4) and Maracanã (-0.3). There were no significant low-low animal clusters.

## Discussion

In this study, we could analyze and compare human and animal sporotrichosis occurrence in Rio de Janeiro Metropolitan Area using official data that is yet under-investigated. In the eight years studied, 7,675 confirmed cases of human sporotrichosis and 3,795 confirmed cases of animal sporotrichosis occurred, accounting for more than 10,000 cases analyzed here, representing only the ones that were completely reported, investigated, and confirmed. Silva et al. ^44^ described 1,848 human sporotrichosis cases in the state of Rio de Janeiro between 1997 and 2007; in this study, we found four times more confirmed cases during the eight years (2013-2020) only in Rio de Janeiro Metropolitan Area.

Temporal patterns in sporotrichosis notifications appear to be influenced by events promoting passive surveillance. Although a technical note mandating notification for humans and animals in Rio de Janeiro was published in 2011 ^45^, the resolution for mandatory notification in humans was only published in June 2013 ^46^. The peak in human sporotrichosis cases occurred in August 2013, just before the resolution was published. Subsequently, case numbers sharply declined from September 2013 through 2014, potentially reflecting initial underreporting as noted by Falcão et al. ^20^. In this study, we observed a similar situation occurring with animal cases reporting. Following the publication of an official resolution mandating animal sporotrichosis notification at the end of 2014, there was a sudden increase in reported cases, followed by a significant decrease.

Between 2014 and 2016, there was a notable increase in human sporotrichosis cases, likely reflecting both a genuine rise in occurrences and a more established surveillance dynamic. Around mid-2015 to late 2016, the number of confirmed animal cases mirrored this human trend, indicating a more stable surveillance system with consistent monthly case numbers and confirmation rates. This period coincided with a local educational governmental campaign on sporotrichosis in the city of Rio de Janeiro, which increased veterinary consultations for suspected cases. Following a decline in confirmed cases in 2018, there was a resurgence in 2019, driven by the expansion of public veterinary services and the launch of an online reporting form by the city government ^47^. However, this increase did not parallel the human trend. Subsequently, there was a sudden decrease in reported cases per month, underscoring the sensitivity of animal sporotrichosis surveillance to initiatives that facilitate notification and encourage public veterinary care-seeking behaviors.

Throughout the time series of human sporotrichosis cases, the proportion of reported and confirmed cases remains consistent. In contrast, for animal sporotrichosis, there is considerable variability in the percentage of confirmed reported cases, particularly from 2017 to 2020. This variability likely indicates the under-confirmation of cases, given the historical link between human and animal sporotrichosis in Rio de Janeiro due to zoonotic transmission ^44^ ^,^ ^48^. The reliability of human surveillance appears notably higher compared to animals, highlighting limitations on how domestic animal surveillance is held. Primarily, the current reporting method for animal cases using SINAN’s epizootic form is inadequate for diseases like sporotrichosis. For example, unlike the form for humans, this system does not allow updates on diagnostic status based on clinical and epidemiological criteria; it only records results from laboratory exams. It is known that correctly diagnosing sporotrichosis is a challenge because of technical difficulties in isolating the fungus ^49^. The absence of more details about the diagnostic results in the loss of important information for animal sporotrichosis surveillance.

Some studies propose alternative reporting methods for human sporotrichosis that can better define transmission dynamics, including questions about interactions with infected animals ^50^. In 2019, the SES/RJ developed a specific form for reporting human sporotrichosis, which was integrated into the Brazilian Ministry of Health digital platform (FormSUS). However, the platform was discontinued in 2021. This form included detailed information that helped understand the relation between animal and human cases, such as identifying if there was animal-related trauma or contact. Despite these advancements, the forms primarily prioritized human notification, with animal surveillance remaining a secondary concern. Moreover, only improving the notification tool will not resolve underreporting; it is necessary to train professionals, especially veterinarians, on how to report suspected cases to optimize form completion and improve the quality of information.

Spatial patterns of human sporotrichosis in Rio de Janeiro Metropolitan Area reveal higher incidence in neighborhoods northwest of the capital and bordering subdistricts. This pattern was first observed in human cases between 1997 and 2007 and named “sporotrichosis belt” ^44^. We could acknowledge this belt has expanded over the years to include other municipalities in Rio de Janeiro Metropolitan Area and additional neighborhoods within the capital, like favelas in the southern area like Rocinha and Vidigal. This expansion was already described between 2008 and 2015 ^20^, and is highlighted here by including 2019-2020. We found a positive correlation in the spatial distribution of human sporotrichosis incidence, which is expected, considering that transmission is related to infected cats, animals that can walk long distances infecting other animals and people inside their home range ^51^ ^,^ ^52^. However, sporotrichosis expansion is not related only to space proximity, because even as sporotrichosis has reached wealthier areas in Rio de Janeiro Metropolitan Area, particularly in the southern part of the capital, it remains predominantly concentrated in neglected areas like *favelas*.

Clusters of high human sporotrichosis incidence have been noted in neighborhoods west of the capital, as well as in municipalities outside the traditional sporotrichosis belt in the northwest of Rio de Janeiro Metropolitan Area. This indicates that the hyperendemic has the potential to spread more extensively throughout Rio de Janeiro Metropolitan Area and possibly beyond, mirroring trends observed in other parts of the state ^20^. Although the northeast of Rio de Janeiro Metropolitan Area continues to present low sporotrichosis incidences, high values observed in southeast subdistricts evidence the need for attention. Additionally, clusters of low incidences of sporotrichosis were also observed in rich areas and areas with lower human density, specifically in the southeast and southwest of the city of Rio de Janeiro, respectively.

Although the animal sporotrichosis ratio per inhabitants was also correlated to space, the magnitude of the correlation was lower than for the incidence of human cases. A study in a municipality of São Paulo has shown strong spatial correlation between feline sporotrichosis cases, but the city had an apparently well-structured surveillance program for animal sporotrichosis ^53^. High animal ratios in the west and north of the city of Rio de Janeiro follow the same patterns observed regarding human incidence, and both show a similar cluster for high values in the western neighborhoods of the city. However, the same phenomenon observed in the time series is observed in spatial analysis: values related to animal cases show high variability.

The spatial pattern of the ratios of animal cases appears to correlate with the presence of public veterinary establishments. In the city of Rio de Janeiro, there are four of these establishments located in Praça da Bandeira and Manguinhos (east), and in Santa Cruz and Guaratiba (west). These areas have high values for the ratio of animal cases, and in Praça da Bandeira there is even an extreme outlier. This suggests that in Rio de Janeiro Metropolitan Area, surveillance of animal sporotrichosis reflects the availability of public veterinary services rather than the true distribution of the disease. In contrast, in southern Brazil, where there is no specific disease control program, higher incidences of feline sporotrichosis in the richest neighborhoods have been linked to easier access to diagnostics at private veterinary clinics ^29^. Moreover, the deficiency of public veterinary care in Rio de Janeiro as a direct challenge to combating sporotrichosis was pointed out years ago ^7^. Despite the four public veterinary facilities in the capital city, their services and free treatment availability for sporotrichosis are often not widely known among the general population ^54^.

Additionally, the spatial pattern of animal ratios differs between reported cases and confirmed cases, consistent with observations in the time series. This disparity is particularly evident in Maricá (southeast) and Duque de Caxias (central north). Given the high incidences of human sporotrichosis in these areas, it is unlikely that animal cases are being overreported without subsequent confirmation. Instead, we hypothesize that the under confirmation of cases is primarily due to limitations in case investigation and diagnostics, potentially influenced by the design constraints of SINAN’s epizootic form, as discussed earlier.

Spatial proximity to feline sporotrichosis cases has been proven to be a risk factor for the disease in humans and other cats ^30^. Thus, areas where human incidence and animal ratios differ too much must be carefully investigated. Our findings show that there are areas where human cases are occurring, but no or few animal cases are being reported. Furthermore, they may not even be diagnosed or treated, which may reflect the seriousness of the situation in these areas. This highlights the limitations of the current sporotrichosis surveillance: some areas are reporting abundant human cases but few animal cases, and these locations should be prioritized for active surveillance strategies ^28^, to find infected individuals (humans and animals) that are not being accounted for or treated.

Animal sporotrichosis cases analyzed here included both cats and dogs. Cats are more susceptible to *Sporothrix* sp. than other animals ^4^ ^,^ ^55^, so it is expected that they account for most cases. However, more than 1,000 suspected sporotrichosis cases were reported in dogs during the studied period. Although there isn’t enough evidence to prove that zoonotic infection via infected dogs is epidemiologically relevant, canine cases can indicate the presence of infected cats because they are probably being contaminated by them ^56^ ^,^ ^57^. Figueiredo et al. ^23^ showed that canine sporotrichosis had a spatial expansion concomitant to human cases in Rio de Janeiro Metropolitan Area, highlighting the importance of monitoring dog cases alongside cats and humans.

Although sporotrichosis surveillance is fragmented in a zoonotic context, with human and animal dimensions assessed separately. Analyzing both together helped us hypothesize possible underreporting of cases. We observed that surveillance for animal sporotrichosis is lacking, which is worrying given that the primary challenges associated with this disease revolve around controlling and treating infected cats ^3^ ^,^ ^29^ ^,^ ^30^ ^,^ ^58^. While national mandatory notification for human sporotrichosis is needed ^58^, a continuous surveillance program for animal sporotrichosis in endemic areas is crucial ^25^. Hence, this emphasizes the importance of a One Health approach for combating sporotrichosis, i.e. surveillance and control strategies must be developed considering humans, animals and the environment simultaneously ^3^ ^,^ ^6^. Focusing solely on human or animal cases could overlook critical areas that are key to effectively address the issue.

Despite the insights provided by this research, some limitations should be considered. We analyzed secondary data from Brazil’s surveillance system, which brought limitations related to missing information, underreporting and a challenging interpretation of results. We couldn’t calculate animal case incidence due to unreliable population estimates, so we opted for an alternative strategy for calculating animal ratios in relation to the human population. Moreover, the results presented here are primarily descriptive but provide an important update about human and animal sporotrichosis in Rio de Janeiro Metropolitan Area and its current surveillance.

## References

[1] Barros MBL, Paes RA, Schubach AO (2011). Sporothrix schenckii and sporotrichosis. Clin Microbiol Rev.

[2] Pereira SA, Gremião IDF, Kitada AAB, Boechat JS, Viana PG, Schubach TMP (2014). The epidemiological scenario of feline sporotrichosis in Rio de Janeiro, State of RJ, Brazil. Rev Soc Bras Med Trop.

[3] Rossow JA, Queiroz-Telles F, Caceres DH, Beer KD, Jackson BR, Pereira JG (2020). A one health approach to combatting Sporothrix brasiliensis narrative review of an emerging zoonotic fungal pathogen in South America. J Fungi (Basel).

[4] Gremião IDF, Miranda LHM, Reis EG, Rodrigues AM, Pereira SA (2017). Zoonotic epidemic of sporotrichosis cat to human transmission. PLoS Pathog.

[5] Lecca LO, Paiva MT, de Oliveira CSF, Morais MHF, de Azevedo MI, Bastos CV (2020). Associated factors and spatial patterns of the epidemic sporotrichosis in a high density human populated area a cross-sectional study from 2016 to 2018. Prev Vet Med.

[6] Gremião IDF, Oliveira MME, Monteiro de Miranda LH, Freitas DFS, Pereira SA (2020). Geographic expansion of sporotrichosis, Brazil.. Emerg Infect Dis.

[7] Barros MBL, Schubach TP, Coll JO, Gremião IDF, Wanke B, Schubach AO (2010). Esporotricose a evolução e os desafios de uma epidemia. Rev Panam Salud Pública.

[8] Rodrigues AM, Della Terra PP, Gremião ID, Pereira SA, Orofino-Costa R, de Camargo ZP (2020). The threat of emerging and re-emerging pathogenic Sporothrix species. Mycopathologia.

[9] Andrade EHP, Moreira SM, Paiva MT, Zibaoui HM, Salvato LA, Azevedo MI (2021). Characterization of animal sporotrichosis in a highly urbanized area. Comp Immunol Microbiol Infect Dis.

[10] Bento AO, Costa ASS, Lima SL, Alves MM, Melo ASA, Rodrigues AM (2021). The spread of cat-transmitted sporotrichosis due to Sporothrix brasiliensis in Brazil towards the Northeast region. PLoS Negl Trop Dis.

[11] Poester VR, Mattei AS, Madrid IM, Pereira JTB, Klafke GB, Sanchotene KO (2018). Sporotrichosis in Southern Brazil, towards an epidemic. Zoonoses Public Health.

[12] Montenegro H, Rodrigues AM, Dias MAG, da Silva EA, Bernardi F, de Camargo ZP (2014). Feline sporotrichosis due to Sporothrix brasiliensis an emerging animal infection in São Paulo, Brazil. BMC Vet Res.

[13] Rocha ICB, Terra PPD, Oliveira RC, Zanotti RL, Falqueto A, Camargo ZP (2021). Molecular-based assessment of diversity and population structure of Sporothrix spp clinical isolates from Espírito Santo-Brazil. Mycoses.

[14] Silva CE, Valeriano CA, Ferraz CE, Neves RP, Oliveira MM, Silva JC (2021). Epidemiological features and geographical expansion of sporotrichosis in the state of Pernambuco, northeastern Brazil. Future Microbiol.

[15] Bernardes-Engemann AR, Almeida MA, Bison I, Rabello VBS, Ramos MLM, Pereira SA (2022). Anti-sporothrix antibody detection in domestic cats as an indicator of a possible new occurrence area for sporotrichosis in North Brazil. Mycopathologia.

[16] Eudes J, Santos IB, Reis CMS, Patané JSL, Paredes V, Bernardes JPRA (2020). A novel Sporothrix brasiliensis genomic variant in Midwestern Brazil: evidence for an older and wider sporotrichosis epidemic.. Emerg Microbes Infect.

[17] Munhoz LS, Poester VR, Severo CB, Trápaga MR, Madrid IM, Benelli JL (2022). Update of the epidemiology of the sporotrichosis epidemic in the state of Rio Grande do Sul, Brazil. Mycoses.

[18] Etchecopaz AN, Lanza N, Toscanini MA, Devoto TB, Pola SJ, Daneri GL (2020). Sporotrichosis caused by Sporothrix brasiliensis in Argentina case report, molecular identification and in vitro susceptibility pattern to antifungal drugs. J Mycol Med.

[19] Rios ME, Suarez J, Moreno J, Vallee J, Moreno JP (2018). Zoonotic sporotrichosis related to cat contact first case report from Panama in Central America. Cureus.

[20] Falcão EMM, Romão AR, Magalhães MAFM, de Lima JB, do Valle ACF, Bastos FI (2022). A spatial analysis of the spread of hyperendemic sporotrichosis in the state of Rio de Janeiro, Brazil.. J Fungi (Basel).

[21] Alzuguir CLC, Pereira SA, Magalhães MAFM, Almeida-Paes R, Freitas DFS, Oliveira LFA (2020). Geo-epidemiology and socioeconomic aspects of human sporotrichosis in the municipality of Duque de Caxias, Rio de Janeiro, Brazil, between 2007 and 2016. Trans R Soc Trop Med Hyg.

[22] Falcão EMM, Lima JB, Campos DP, Valle ACF, Bastos FI, Gutierrez-Galhardo MC (2019). Hospitalizações e óbitos relacionados à esporotricose no Brasil (1992-2015).. Cad Saúde Pública.

[23] Figueiredo ABF, Magalhães MAFM, Tassinari WS, Gremião IDF, Miranda LHM, Menezes RC (2022). Spatial distribution of canine sporotrichosis in Rio de Janeiro, Brazil (1998-2018) and its correlation with socioeconomic conditions. J Fungi (Basel).

[24] Boechat JS, Oliveira MME, Almeida-Paes R, Gremião IDF, Machado ACS, Oliveira RVC (2018). Feline sporotrichosis associations between clinical-epidemiological profiles and phenotypic-genotypic characteristics of the etiological agents in the Rio de Janeiro epizootic area. Mem Inst Oswaldo Cruz.

[25] Sanchotene KO, Madrid IM, Klafke GB, Bergamashi M, Terra PPD, Rodrigues AM (2015). Sporothrix brasiliensis outbreaks and the rapid emergence of feline sporotrichosis. Mycoses.

[26] Machado D, Bragança A, Travnik I, Rossi A, Sant'Anna A (2021). Should cats be allowed outdoors? A research survey on animal welfare risks for free-ranging cats in Brazil.. Anim Welf.

[27] Gremião IDF, Menezes RC, Schubach TMP, Figueiredo ABF, Cavalcanti MCH, Pereira SA (2015). Feline sporotrichosis epidemiological and clinical aspects. Med Mycol.

[28] Moreira SM, Andrade EHP, Paiva MT, Zibaoui HM, Salvato LA, Azevedo MI (2021). Implementation of an animal sporotrichosis surveillance and control program, Southeastern Brazil. Emerg Infect Dis.

[29] Poester VR, Stevens DA, Basso RP, Munhoz LS, Zanchi M, Benelli JL (2022). CATastrophe response to the challenges of zoonotic sporotrichosis in southern Brazil. Mycoses.

[30] Paiva MT, Oliveira CSF, Nicolino RR, Bastos CV, Lecca LO, Azevedo MI (2020). Spatial association between sporotrichosis in cats and in human during a Brazilian epidemics. Prev Vet Med.

[31] Rio de Janeiro (2002). Lei Complementar nº 105, de 4 de julho de 2002.. Diário Oficial do Estado do Rio de Janeiro.

[32] Fórum Nacional de Entidades Metropolitanas Região Metropolitana do Rio de Janeiro (RJ). 2018..

[33] City Mayors The world's largest urban areas in 2020..

[34] Ljung GM, Box GEP (1978). On a measure of lack of fit in time series models. Biometrika.

[35] Cleveland WS (1981). LOWESS a program for smoothing scatterplots by robust locally weighted regression. Am Stat.

[36] Magalhães MAFM, Matos VP, Medronho RA (2014). Avaliação do dado sobre endereço no Sistema de Informação de Agravos de Notificação utilizando georreferenciamento em nível local de casos de tuberculose por dois métodos no município do Rio de Janeiro. Cad Saúde Colet (Rio J.).

[37] Cetl V, Kliment T, Jogun T (2018). A comparison of address geocoding techniques case study of the city of Zagreb, Croatia. Survey Review.

[38] Araújo VEM, Pinheiro LC, Almeida MCM, Menezes FC, Morais MHF, Reis IA (2013). Relative risk of visceral leishmaniasis in Brazil a spatial analysis in urban area. PLoS Negl Trop Dis.

[39] Anselin L (1995). Local indicators of spatial association LISA. Geogr Anal.

[40] Hyndman R, Athanasopoulos G, Caceres G, Chhay L, O'Hara-Wild M, Petropoulos F forecast: forecasting functions for time series and linear models..

[41] Pereira RHM, Goncalves CN geobr: download official spatial data sets of Brazil. R package version 170..

[42] Pebesma E, Bivand R (2023). Spatial sata science: with applications in R.

[43] Bivand R (2022). R packages for analyzing spatial data a comparative case study with areal data. Geogr Anal.

[44] Silva MBT, Costa MMM, Torres CCS, Galhardo MCG, Valle ACF, Magalhães MAFM (2012). Esporotricose urbana epidemia negligenciada no Rio de Janeiro, Brasil. Cad Saúde Pública.

[45] Secretaria de Estado de Saúde Nota Técnica nº 3/2011-GDTVZ/DTI/CVE/SVEA/SVS-SESRJ e IPEC/FIOCRUZ..

[46] Secretaria de Estado de Saúde (2013). Resolução SES nº 674, de 12 de julho de 2013.. Diário Oficial do Estado do Rio de Janeiro.

[47] Prefeitura do Rio de Janeiro Prefeitura amplia em 100% atendimentos a animais nas unidades de zoonoses da Vigilância Sanitária..

[48] Barros MBL, Schubach AO, Valle ACF, Galhardo MCG, Conceição-Silva F, Schubach TMP (2004). Cat-transmitted sporotrichosis epidemic in Rio de Janeiro, Brazil description of a series of cases. Clin Infect Dis.

[49] Conceição-Silva F, Morgado FN (2018). Immunopathogenesis of human sporotrichosis what we already know. J Fungi (Basel).

[50] Garcia MTP, Lima EFA, Leite FMC (2022). Elaboration and evaluation of a compulsory notification form for human sporotrichosis. Esc Anna Nery (Online).

[51] Ferreira JP, Leitão I, Santos-Reis M, Revilla E (2011). Human-related factors regulate the spatial ecology of domestic cats in sensitive areas for conservation. PLoS One.

[52] Macêdo-Sales PA, Souto SRLS, Destefani CA, Lucena RP, Machado RLD, Pinto MR (2018). Domestic feline contribution in the transmission of Sporothrix in Rio de Janeiro State, Brazil a comparison between infected and non-infected populations. BMC Vet Res.

[53] Scuarcialupi LN, Pereira FC, Baquero OS (2021). Feline sporotrichosis social vulnerability and prioritization of geographic areas in Guarulhos, SP, Brazil. Braz J Vet Res Anim Sci.

[54] Papa MGO, Oliveira MH, Reis LLM, Camera PO, Silva ACR (2018). Avaliação do conhecimento dos moradores da Zona Norte do Rio de Janeiro em relação à esporotricose. Revista Brasileira de Educação e Saúde.

[55] Orofino-Costa R, Macedo PM, Rodrigues AM, Bernardes-Engemann AR (2017). Sporotrichosis an update on epidemiology, etiopathogenesis, laboratory and clinical therapeutics. An Bras Dermatol.

[56] Schubach TMP, Schubach A, Okamoto T, Barros MBL, Figueiredo FB, Cuzzi T (2006). Canine sporotrichosis in Rio de Janeiro, Brazil clinical presentation, laboratory diagnosis and therapeutic response in 44 cases (1998-2003). Med Mycol.

[57] Zager Â, Santos LA, Malegoni ACS, Roque LZ, Silva TB, Risso FB (2021). Canine sporotricosis clinic, epidemiology, diagnosis and treatment. OALib.

[58] Gremião IDF, Rocha EMS, Montenegro H, Carneiro AJB, Xavier MO, Farias MR (2021). Guideline for the management of feline sporotrichosis caused by Sporothrix brasiliensis and literature revision. Braz J Microbiol.

